# Metabolites related to gut bacterial metabolism, peroxisome proliferator-activated receptor-alpha activation, and insulin sensitivity are associated with physical function in functionally-limited older adults

**DOI:** 10.1111/acel.12251

**Published:** 2014-07-18

**Authors:** Michael S Lustgarten, Lori L Price, Angela Chalé, Roger A Fielding

**Affiliations:** 1 Nutrition, Exercise Physiology, and Sarcopenia Laboratory, Jean Mayer USDA Human Nutrition Research Center, Tufts University711 Washington Street, Boston, MA, 02111, USA; 2 The Institute for Clinical Research and Health Policy Studies, Tufts Medical Center, Tufts Clinical and Translational Science Institute, Tufts University711 Washington Street, Boston, MA, 02111, USA

**Keywords:** aging, gut bacterial metabolites, insulin sensitivity, metabolomics, peroxisome proliferator-activated receptor-alpha, physical function

## Abstract

Identification of mechanisms underlying physical function will be important for addressing the growing challenge that health care will face with physical disablement in the expanding aging population. Therefore, the goals of the current study were to use metabolic profiling to provide insight into biologic mechanisms that may underlie physical function by examining the association between baseline and the 6-month change in serum mass spectrometry-obtained amino acids, fatty acids, and acylcarnitines with baseline and the 6-month change in muscle strength (leg press one repetition maximum divided by total lean mass, LP/Lean), lower extremity function [short physical performance battery (SPPB)], and mobility (400 m gait speed, 400-m), in response to 6 months of a combined resistance exercise and nutritional supplementation (whey protein or placebo) intervention in functionally-limited older adults (SPPB ≤ 10; 70–85 years, *N* = 73). Metabolites related to gut bacterial metabolism (cinnamoylglycine, phenol sulfate, p-cresol sulfate, 3-indoxyl sulfate, serotonin, *N*-methylproline, hydrocinnamate, dimethylglycine, trans-urocanate, valerate) that are altered in response to peroxisome proliferator-activated receptor-alpha (PPAR-α) activation (α-hydroxyisocaproate, α-hydroxyisovalerate, 2-hydroxy-3-methylvalerate, indolelactate, serotonin, 2-hydroxypalmitate, glutarylcarnitine, isobutyrylcarnitine, cinnamoylglycine) and that are related to insulin sensitivity (monounsaturated fatty acids: 5-dodecenoate, myristoleate, palmitoleate; γ-glutamylamino acids: γ-glutamylglutamine, γ-glutamylalanine, γ-glutamylmethionine, γ-glutamyltyrosine; branched-chain amino acids: leucine, isoleucine, valine) were associated with function at baseline, with the 6-month change in function or were identified in backward elimination regression predictive models. Collectively, these data suggest that gut microbial metabolism, PPAR-α activation, and insulin sensitivity may be involved in mechanisms that underlie physical function in functionally-limited older adults.

## Introduction

Disability, defined as a limited ability to perform regular activities of daily living (Jette, 2006), increases with advancing age (Launer *et al*., 1994) and is predictive of future hospitalization, institutionalization, and mortality (Harris *et al*., 1989), thereby placing an increased demand on the healthcare system. Skeletal muscle strength, lower extremity function, and mobility are each associated with disability risk in older adults (Guralnik *et al*., 1994; Newman *et al*., 2006; Puthoff & Nielsen, 2007). For example, muscle strength, measured with the bilateral leg press one repetition maximum (LP), is associated with performance on the late life function and disability index (Puthoff & Nielsen, 2007). As a measure of lower extremity function, performance on the short physical performance battery (SPPB) independently predicts mobility disability and activities of daily living disability (Guralnik *et al*., 1994). Mobility, defined as the ability to walk safely and independently, is measured by the time needed to walk 400 m (400-m gait speed; 400-m) (Heffernan *et al*., 2012). Each additional minute needed to complete the 400-m test is associated with increased risk for mobility limitation, disability, and incident cardiovascular disease (Newman *et al*., 2006). Furthermore, decreased muscle strength, SPPB, and 400-m performance are associated with an increased risk for all-cause mortality (Guralnik *et al*., 1994; Newman *et al*., 2006; Ruiz *et al*., 2008). Therefore, identification of mechanisms underlying muscle strength, lower extremity function, and mobility will be important for addressing the growing challenge that health care will face with physical disablement in the expanding aging population.

One approach that can be used to elucidate mechanisms related to physical function in older adults is mass spectrometry (MS)-based metabolomics, which aims to characterize and quantify all of the metabolites in a biological sample, thereby providing an analytical description of complex metabolic processes (Fiehn, 2002). For example, MS-based metabolomic profiling of amino acids, fatty acids, and acylcarnitines has provided support for the hypothesis that lipid-induced insulin resistance is explained in part by an overload of mitochondrial lipid oxidation, thereby resulting in mitochondrial stress, reduced oxidative capacity, and activation of signaling pathways that interfere with insulin action (Newgard *et al*., 2009). Although diminished oxidative capacity is associated with reduced physical function (Coen *et al*., 2013), to date, only one study has investigated the association between amino acids, fatty acids, or acylcarnitines with physical function in older adults, as plasma acylcarnitines were negatively associated with the SPPB and with maximal gait speed (Lum *et al*., 2011). Furthermore, the metabolomic response to the 6-month change in function as a result of a combined resistance exercise and nutritional supplementation (whey protein or placebo) intervention in older adults has yet to be studied. Therefore, the goals of the current study were to characterize the association between baseline and the 6-month change in amino acids, fatty acids, and acylcarnitines with baseline and the 6-month change in physical function as a result of a combined resistance exercise and nutritional supplementation intervention, in functionally-limited older adults.

## Results

Results for the LP one repetition maximum, SPPB, 400-m, total lean and fat mass, BMI, average dietary protein and fiber intake in older adults (mean age, 77.7 ± 3.9 years; *N* = 73, 43 women, 30 men) from the randomized, double-blind, controlled study of (Chale *et al*., 2012) were used. It is important to note that although 6 months of resistance exercise training significantly maintained or improved function when compared with function at baseline, whey protein supplementation was not found to further improve physical function when compared with placebo (Chale *et al*., 2012). To account for the positive association between the LP one repetition maximum with total lean mass (*r* = 0.71, *P* < 0.0001), LP was divided by total lean mass (LP/Lean). Average values at baseline and after 6 months of the combined resistance exercise and nutritional supplementation intervention for LP/Lean were 26.9 ± 5.9 N kg^−1^, 31.5 ± 7.3 N kg^−1^ (*P* < 0.05); SPPB, 8.5 ± 1.5, 10.3 ± 1.6 (*P* < 0.05); 400-m, 1.0 ± 0.2 m s^−1^, 1.1 ± 0.2 m s^−1^; total fat mass, 25.6 ± 7.2 kg, 25.4 ± 6.7 kg; BMI, 27.0 ± 3.2 kg m^−2^, 27.7 ± 3.1 kg m^−2^ (*P* < 0.05); dietary protein intake, 69.7 ± 17.4 g, 78.1 ± 23.5 g (*P* < 0.05); dietary fiber intake, 19.2 ± 8.2 g, 15.9 ± 6.9 (*P* < 0.05). In addition, the 6-month change in serum creatinine was not significantly different when compared with baseline values (0.9 ± 0.3, 0.9 ± 0.3, *P* = 0.45).

### Principal components analysis factors associated with LP/Lean, SPPB, and 400-m

Significant and nonsignificant associations between principal components analysis (PCA) factors with LP/Lean, SPPB, and 400-m at baseline are shown in Table 1 and Table S1 (Supporting information), respectively. FA Factor 9 (2-hydroxypalmitate, 2-hydroxystearate) was positively associated, whereas AA Factor 6 (cinnamoylglycine, hydrocinnamate) was negatively associated with LP/Lean. The significant associations identified between FA Factor 9 and AA Factor 6 with LP/Lean remained when examined for their association with LP, after adjusting for sex, age, total fat mass, and total lean mass (FA Factor 9, β ± SE, 68.8 ± 32.2, *P* = 0.04; AA Factor 6, β ± SE, −70.5 ± 32.4, *P* = 0.03).

**Table 1:** Principal components analysis (PCA) factors associated with LP/Lean, short physical performance battery (SPPB), and 400-m at baseline

	Component loadings	β ± SE	*P*-value	*q*-value
LP/Lean				
FA Factor 9: 2-hydroxypalmitate, 2-hydroxystearate	0.90, 0.42	1.5 ± 0.7	0.03	0.25
AA Factor 6: Cinnamoylglycine, hydrocinnamate	0.91, 0.88	−1.5 ± 0.7	0.04	0.25
Dipeptide Factor 3: γ-glutamylamino acids (glutamine, alanine, methionine)	0.91, 0.81, 0.74	−1.2 ± 0.7	0.09	0.29
AA Factor 19: *N*-acetylglycine	0.92	1.2 ± 0.7	0.09	0.29
SPPB				
AA Factor 6: Cinnamoylglycine, hydrocinnamate	0.91, 0.88	−0.5 ± 0.2	0.009	0.25
AA Factor 7: *N*,*N*-dimethylproline, *N*-methylproline	0.92, 0.91	−0.4 ± 0.2	0.02	0.25
400-m				
AA Factor 19: *N*-acetylglycine	0.92	0.1 ± 0.0	0.009	0.25
AA Factor 4: α-hydroxyisovalerate, 2-hydroxy-3-methylvalerate, α-hydroxyisocaproate, indolelactate	0.87, 0.78, 0.78, 0.67	0.1 ± 0.0	0.02	0.25
AC Factor 4: Isobutyrylcarnitine		−0.0 ± 0.0	0.02	0.25
FA Factor 7: C12, C14, C16 Unsaturated FA’s	0.79, 0.78, 0.48	0.0 ± 0.0	0.03	0.25
AA Factor 5: Valine, Leucine, Isoleucine	0.80, 0.77, 0.76	−0.0 ± 0.0	0.04	0.25
AA Factor 12: Phenol Sulfate	0.91	−0.0 ± 0.0	0.04	0.25
AA Factor 13: Trans-urocanate	0.92	−0.0 ± 0.0	0.05	0.25
AA Factor 1: 2-hydroxyisobutyrate, *N*-acetylthreonine, urea, C-glytryptophan, *N*-acetylalanine, *N*-formylmethionine, β-hydroxyisovalerate, N_6_-acetyllysine, serine (-)	0.90, 0.79, 0.74, 0.70, 0.58, 0.48, 0.44, 0.42, −0.46	−0.0 ± 0.0	0.06	0.29
AC Factor 5: Tiglyl carnitine	0.92	0.0 ± 0.0	0.09	0.29
FA Factor 13: C11 Unsaturated FA	0.92	0.0 ± 0.0	0.09	0.29

LP/Lean, leg press one repetition maximum divided by total lean mass.

Sex, age, total fat (LP/Lean model) or BMI (SPPB, 400-m models)-adjusted associations between PCA factors with baseline LP/Lean, SPPB, and 400-m are shown with component loadings for metabolites contained within each respective PCA factor, parameter estimates and standard errors (β ± SE), in order of significance (*P*-value), and with *q*-values.

AA Factor 6 (cinnamoylglycine, hydrocinnamate) and AA Factor 7 (*N*,*N*-dimethylproline, *N*-methylproline) were negatively associated with the SPPB.

AA Factor 19 (*N*-acetylglycine), AA Factor 4 (α-hydroxyisovalerate, 2-hydroxy-3-methylvalerate, α-hydroxyisocaproate, indolelactate), and FA Factor 7 (monounsaturated fatty acids (MUFA): 5-dodecenoate, myristoleate, palmitoleate) were positively associated, whereas AC Factor 4 (isobutyrylcarnitine), AA Factor 5 [branched-chain amino acids (BCAA): leucine, isoleucine, valine], AA Factor 12 (phenol sulfate), and AA Factor 13 (trans-urocanate) were negatively associated with 400-m.

Inclusion of covariates with *P*-values <0.05 in regression-based predictive modeling can fail in identifying variables known to be important (Mickey & Greenland, 1989). Therefore, PCA factors that were associated with measures of physical function at 0.05 ≤ *P* ≤ 0.10 [as recommended by Bursac *et al*. (2008)] and *q* ≤ 0.30 are shown in Table 1 and were additionally considered as candidate variables for backward elimination (BE) linear regression. BE models for LP/Lean, SPPB, and 400-m are shown in Table 2. Whereas sex and age explained 6% of the variance (adjusted *R*^2^) inherent in LP/Lean, the combination of AA Factors 6 and 19, FA Factor 9, and Dipeptide Factor 3 explained an additional 16%, for a total adjusted *R*^2^ of 0.22 (model *P* = 0.0009). Separately, the combination of AA Factors 6 and 7 explained 15% of the variance inherent in the SPPB (model *P* = 0.001). Whereas sex and age explained 7% of the variance inherent in 400-m, the combination of AA Factors 4, 5, 12, 13, and 19, and AC Factor 4 explained an additional 34%, for a total adjusted *R*^2^ of 0.41 (model *P* < 0.0001).

**Table 2:** LP/Lean, short physical performance battery (SPPB), and 400-m backward elimination (BE) regression models at baseline

	β ± SE	*P*-value
LP/Lean: Adjusted *R*^2^ = 0.22, *P* = 0.0009		
Sex	4.2 ± 1.4	0.003
FA Factor 9: 2-hydroxypalmitate, 2-hydroxystearate	1.6 ± 0.6	0.01
AA Factor 6: Hydrocinnamate, cinnamoylglycine	−1.4 ± 0.6	0.03
Age	−0.4 ± 0.2	0.05
Dipeptide Factor 3: γ-glutamylamino acids	−1.3 ± 0.6	0.05
AA Factor 19: *N*-acetylglycine	1.2 ± 0.6	0.07
SPPB: Adjusted *R*^2^ = 0.15, *P* = 0.001		
AA Factor 6: Hydrocinnamate, cinnamoylglycine	−0.4 ± 0.2	0.008
AA Factor 7: *N*-methylproline, *N*,*N*-dimethylproline	−0.4 ± 0.2	0.009
400-m: Adjusted *R*^2^ = 0.41, *P* < 0.0001		
Age	−0.0 ± 0.0	0.0005
AA Factor 19: *N*-acetylglycine	0.1 ± 0.0	0.0006
AC Factor 4: Isobutyrylcarnitine	−0.1 ± 0.0	0.004
AA Factor 4: BCAA degradation products, indolelactate	0.0 ± 0.0	0.005
AA Factor 12: Phenol Sulfate	−0.1 ± 0.0	0.009
Sex	0.1 ± 0.0	0.01
AA Factor 5: BCAA	−0.0 ± 0.0	0.02
AA Factor 13: Trans-urocanate	−0.0 ± 0.0	0.08

BCAA, branched-chain amino acids; LP/Lean, leg press one repetition maximum divided by total lean mass.

Sex, age, total fat (LP/Lean model) or BMI (SPPB, 400-m models), and principal components analysis factors that were associated (*P* ≤ 0.10 and *q* ≤ 0.30) with measures of physical function were considered as candidate variables for BE linear regression. Covariates significantly associated (*P* ≤ 0.10) with LP/Lean, SPPB, and 400-m are shown with parameter estimates and standard errors (β ± SE), in order of significance (*P*-value).

Metabolites that have been previously found in the serum or colonic lumen from conventional, but not germ-free mice (Wikoff *et al*., 2009; Matsumoto *et al*., 2012) and that were contained within PCA factors that were significantly associated with measures of function or that were identified in BE models, including AA Factors 6, 7, 12, and 13 were examined for their association with average dietary protein intake and serum creatinine, but significant positive associations were not identified (Table S2, Supporting information). Separately, *N*-acetylglycine was significantly associated with average dietary fiber intake, after adjusting for sex, age, and BMI (β ± SE, 2.1 ± 1.0, *P* = 0.03).

### Six-month change in principal components analysis factors associated with the 6-month change in LP/Lean, SPPB, and 400-m

Significant and nonsignificant associations between the 6-month change in PCA factors with the 6-month change in LP/Lean, SPPB, and 400-m are shown in Table 3 and Tables S3 and S4 (Supporting information), respectively. The 6-month change in AC Factor 4 (glutarylcarnitine) was positively associated, whereas the 6-month change in AA Factor 13 (N_6_-acetyllysine) was negatively associated with the 6-month change in LP/Lean. Although the significant association identified between the 6-month change in AC Factor 4 with the 6-month change in LP remained (β ± SE, 81.6 ± 10.8, *P* = 0.05) after adjustment for sex, age, the 6-month change in total lean mass, the 6-month change in total fat mass, whey/placebo group designation and baseline LP, the association between the 6-month change in AA Factor 13 with the 6-month change in LP was not significant (β ± SE, −63.5 ± 40.8, *P* = 0.13).

**Table 3:** Six-month change in principal components analysis (PCA) factors associated with the 6-month change in LP/Lean, short physical performance battery (SPPB), and 400-m

	Component loadings	β ± SE	*P*-value	*q*-value
Six-month change in LP/Lean				
AC Factor 4: Glutarylcarnitine	0.94	1.5 ± 0.7	0.05	0.25
AA Factor 13: N_6_-acetyllysine	0.90	−1.4 ± 0.7	0.05	0.25
Dipeptide Factor 1: γ-glutamyBCAAs (leucine, isoleucine, valine), leucylleucine	0.90, 0.88, 0.84, 0.41	−1.4 ± 0.7	0.07	0.29
FA Factor 5: Valerate	0.94	1.3 ± 0.7	0.07	0.29
Dipeptide Factor 8: *N*-acetylmethionine	0.94	−1.3 ± 0.7	0.08	0.29
Six-month change in SPPB				
FA Factor 8: 3-carboxy-4-methyl-5-propyl-2-furanpropionate	0.94	0.4 ± 0.2	0.01	0.25
AA Factor 12: Serotonin	0.95	0.4 ± 0.2	0.03	0.25
Dipeptide Factor 9: γ-glutamyltyrosine	0.92	−0.3 ± 0.2	0.04	0.25
AA Factor 17: Dimethylglycine	0.90	−0.3 ± 0.2	0.05	0.25
AA Factor 2: Glycine, serine, asparagine, alanine, glutamine	0.90, 0.79, 0.71, 0.53, 0.47	0.3 ± 0.2	0.08	0.29
Dipeptide Factor 10: Leucylphenylalanine	0.90	−0.3 ± 0.2	0.09	0.29
AA Factor 14: *N*-acetyl-β-alanine	0.93	−0.3 ± 0.2	0.09	0.29
Six-month change in 400-m				
AA Factor 8: Indoleacetate	0.93	−0.1 ± 0.0	0.03	0.25
AC Factor 5: Isobutyrylcarnitine	0.93	0.1 ± 0.0	0.03	0.25
AA Factor 5: p-cresol sulfate, 3-indoxyl sulfate	0.88, 0.81	0.1 ± 0.0	0.05	0.25

BCAA, branched-chain amino acids; LP/Lean, leg press one repetition maximum divided by total lean mass.

Sex, age, 6-month fat change (LP/Lean model) or 6-month BMI change (SPPB, 400-m models), whey/placebo group designation, and baseline function (for each respective model)-adjusted associations between the 6-month change in PCA factors with the 6-month change in LP/Lean, SPPB, and 400-m are shown with component loadings for metabolites contained within each respective PCA factor, parameter estimates and standard errors (β ± SE), in order of significance (*P*-value), and with *q*-values.

The 6-month change in FA factor 8 (3-carboxy-4-methyl-5-propyl-2-furanpropionate, CMPF) and AA Factor 12 (serotonin) were positively associated, whereas the 6-month change in Dipeptide Factor 9 (γ-glutamyltyrosine) and AA Factor 17 (dimethylglycine) were negatively associated with the 6-month change in SPPB.

The 6-month change in AC Factor 5 (isobutyrylcarnitine) and AA Factor 5 (p-cresol sulfate, 3-indoxyl sulfate) were positively associated, whereas the 6-month change in AA Factor 8 (indoleacetate) was negatively associated with the 6-month change in 400-m.

The 6-month change in PCA factors that were associated with the 6-month change in LP/Lean, SPPB, and 400-m at *P* ≤ 0.10 [as recommended by Bursac *et al*. (2008)] and *q* ≤ 0.30 are shown in Table 3 and were additionally considered as candidate variables for BE regression. BE models for the 6-month change in LP/Lean, SPPB, and 400-m are shown in Table 4. Whereas sex and baseline LP/Lean explained 9% of the variance inherent in the 6-month change in LP/Lean, the combination of the 6-month change in AA Factor 13 and FA Factor 5 explained an additional 8%, for a total adjusted *R*^2^ = 0.17 (model *P* = 0.004). Although baseline SPPB explained 9% of the variance inherent in the 6-month change in SPPB, the combination of the 6-month change in AA Factors 12, 14, and 17, Dipeptide Factors 9 and 10, and FA Factor 8 explained an additional 31%, for a total adjusted *R*^2^ = 0.40 (model *P* < 0.0001). Baseline 400-m explained 19%, and the combination of the 6-month change in AA Factor 8 and AC Factor 5 explained 7% of the variance inherent in the 6-month change in 400-m, for a total adjusted *R*^2^ = 0.26 (model *P* < 0.0001).

**Table 4:** Six-month change in LP/Lean, short physical performance battery (SPPB), and 400-m backward elimination (BE) regression models

	β ± SE	*P*-value
Six-month change in LP/Lean: Adjusted *R*^2^ = 0.17, *P* = 0.004		
Sex	4.4 ± 1.5	0.004
Baseline LP/Lean	−0.3 ± 0.1	0.02
AA Factor 13: N_6_-acetyllysine	−1.4 ± 0.7	0.05
FA Factor 5: Valerate	1.3 ± 0.7	0.07
Six-month change in SPPB: Adjusted *R*^2^ = 0.40, *P* < 0.0001		
AA Factor 14: *N*-acetyl-β-alanine	−0.4 ± 0.1	0.002
AA Factor 12: Serotonin	0.3 ± 0.1	0.01
FA Factor 8: 3-carboxy-4-methyl-5-propyl-2-furanpropionate	0.4 ± 0.1	0.01
AA Factor 17: Dimethylglycine	−0.3 ± 0.1	0.01
Dipeptide Factor 10: Leucylphenylalanine	−0.3 ± 0.1	0.01
Baseline SPPB	−0.2 ± 0.1	0.03
Dipeptide Factor 9: γ-glutamyltyrosine	−0.2 ± 0.1	0.09
Six-month change in 400-m: Adjusted *R*^2^ = 0.26, *P* < 0.0001		
Baseline 400-m	−0.7 ± 0.2	0.0001
AA Factor 8: Indoleacetate	−0.1 ± 0.0	0.04
AC Factor 5: Isobutyrylcarnitine	0.1 ± 0.0	0.06

LP/Lean, leg press one repetition maximum divided by total lean mass.

Sex, age, 6-month fat change (LP/Lean model) or 6-month BMI change (SPPB, 400-m models), whey/placebo group designation, baseline function (for each respective model), and the 6-month change in principal components analysis factors that were associated (*P* ≤ 0.10 and *q* ≤ 0.30) with the 6-month change in measures of physical function were considered as candidate variables for BE linear regression. Covariates significantly associated (*P* ≤ 0.10) with the 6-month change in LP/Lean, SPPB, and 400-m are shown with parameter estimates and standard errors (β ± SE), in order of significance (*P*-value).

Metabolites previously found in the serum or colonic lumen from conventional, but not germ-free mice (Wikoff *et al*., 2009; Matsumoto *et al*., 2012) that were contained within 6-month change PCA factors that were significantly associated with the 6-month change in LP/Lean, SPPB, or 400-m, or, that were identified in BE models, including the 6-month change in FA Factor 5, AA Factors 5, 8, 12, and 17 were examined for their association with the 6-month change in average dietary protein intake and serum creatinine. The 6-month change in average dietary protein intake or serum creatinine was not significantly positively associated with the 6-month change in FA Factor 5 or the 6-month change in AA Factors 5, 8, 12, and 17 (Table S5, Supporting information).

## Discussion

The main findings of the current study are that metabolites related to gut bacterial metabolism, peroxisome proliferator-activated receptor-alpha (PPAR-α) activation, and insulin sensitivity were associated with baseline and the 6-month change in physical function as a result of a combined resistance exercise and nutritional supplementation intervention in functionally-limited older adults.

Cinnamoylglycine, phenol sulfate, p-cresol sulfate, 3-indoxyl sulfate, and serotonin (~threefold increased) and hydrocinnamate, *N*-methylproline, dimethylglycine, trans-urocanate, and valerate are found in serum and the colonic lumen from conventional, but not germ-free mice (Wikoff *et al*., 2009; Matsumoto *et al*., 2012), respectively, evidence that suggests a role for gut bacterial metabolic products on influencing physical function in functionally-limited older adults. Furthermore, colonic bacteria ferment dietary fiber to produce short chain fatty acids, including acetate (Cummings, 1983). *N*-acetylglycine, as the glycine conjugate of acetyl-CoA (Schachter & Taggart, 1954) was positively associated with dietary fiber intake, LP/Lean, and 400-m, evidence that additionally suggests a role for gut bacteria and their related metabolic products on influencing physical function. An increased dietary protein intake may be related to an increase in serum amino acid-related metabolic products (Burke *et al*., 2012), and therefore, we tested these PCA factors for their association with average dietary protein intake, but significant associations were not identified. Separately, the finding that cinnamoylglycine, CMPF, p-cresol sulfate, and 3-indoxyl sulfate accumulate when kidney function is impaired (Niwa, 2011; Boelaert *et al*., 2013) suggests a link between kidney function, accumulation of serum metabolites, and physical function. However, serum creatinine [as a marker of kidney function; (Levey, 1990)] was not significantly associated with any gut bacterial metabolite-related PCA factors. Therefore, we suggest that an altered gut microflora may be responsible for the presence of these metabolites in serum. For example, phenylalanine degradation by *Clostridium* (*C*.) *Sporogenes* produces cinnamic acid (glycine conjugation yields cinnamoylglycine) (Caldwell *et al*., 1976) and hydrocinnamate (Barker, 1981). Furthermore, *Clostridium difficile* and *C. sporogenes* produce isobutryic acid (Mainil, 2006), the metabolic precursor for the formation of isobutyrylcarnitine, and *C. difficile* degrades tryptophan to produce indoleacetate (Brooks *et al*., 1984). Because aging is associated with an increased risk of *C*. *difficile* carriage (McFarland *et al*., 1990), these data collectively suggest a negative role for *Clostridia*-related metabolic products on influencing physical function in older adults. To date, however, only one study has investigated the impact of exercise on gut microbiota, as endurance exercise reduced *Clostridia*, in mice (Choi *et al*., 2013). Collectively, these data suggest that future studies aimed identifying the association between, and, the causative role of gut bacteria and their related metabolic products on influencing physical function in older adults are of interest.

Findings for α-hydroxyisocaproate, α-hydroxyisovalerate, 2-hydroxy-3-methylvalerate, indolelactate, serotonin, 2-hydroxypalmitate, glutarylcarnitine, isobutyrylcarnitine, and cinnamoylglycine suggest a role for PPAR-α activation on influencing physical function in functionally-limited older adults. PPAR-α is a nuclear hormone receptor family transcription factor (Issemann & Green, 1990) that is involved in the transcriptional activation of genes that regulate energy metabolism (Michalik *et al*., 2006), a potentially important finding because mitochondrial oxidative capacity is reduced in association with reduced physical function in older adults (Coen *et al*., 2013). Peroxisome proliferator-activated receptor-alpha mRNA and protein are decreased during aging (Poynter & Daynes, 1998; Sung *et al*., 2004), and PPAR-α^−/−^ mice exhaust faster and run a shorter distance than WT mice (Muoio *et al*., 2002), evidence that may provide a link between age-related decreases in physical function with decreased PPAR-α activation. In support of the hypothesis that PPAR-α activation may play a role in mechanisms underlying physical function, plasma levels of α-hydroxyisocaproate, indolelactate, and serotonin are increased as a result of PPAR-α activation (Ohta *et al*., 2009) and were each positively associated with physical function in the current study. Furthermore, each of the metabolites found in AA Factor 4 (including α-hydroxyisocaproate, α-hydroxyisovalerate, 2-hydroxy-3-methylvalerate, indolelactate) are the reduced version of their corresponding keto-acid, resulting in the consumption of NADH and the production of NAD+. For example, indolepyruvate reduction by NADH produces NAD+ and indolelactate (Manna *et al*., 2010). NADH consumption and the corresponding increase in the NAD+/NADH ratio has been shown to increase PPAR-α expression (Hwang *et al*., 2009), findings that suggest a link between AA Factor 4 with PPAR-α activation. In addition, PPAR-α activation upregulates gene expression of *FA2H*, *CPT*, and *ACAD8* (Rakhshandehroo *et al*., 2007; Makowski *et al*., 2009; Takeda *et al*., 2013), findings that may explain the associations found between 2-hydroxypalmitate, glutarylcarnitine [reductions in glutarylcarnitine are related to levels of CPT (Lee & Wolfgang, 2012)], and isobutyrylcarnitine with physical function. Separately, PPAR-α activation has been shown to reduce urinary excretion of isobutyrylcarnitine (Patterson *et al*., 2009) and cinnamoylglycine (Zhen *et al*., 2007), and cinnamoylglycine is increased in PPAR^−/−^ mice (Zhen *et al*., 2007). Collectively, these data suggest a role for mechanisms related to PPAR-α activation on influencing physical function in functionally-limited older adults.

Associations between MUFA (FA Factor 7, FA Factor 13), γ-glutamylamino acids (Dipeptide Factor 3, 6-month change in Dipeptide Factors 1 and 9), and BCAAs (AA Factor 5) with measures of physical function suggest a role for mechanisms related to insulin sensitivity on influencing physical function. In agreement with this, insulin resistance is associated with reduced exercise capacity (Segerstrom *et al*., 2011). Circulating levels of the MUFA palmitoleate (found in FA Factor 7) strongly and independently predict insulin sensitivity (Stefan *et al*., 2010). Similarly, consumption of dietary MUFA is associated with improved insulin sensitivity (Gillingham *et al*., 2011). In contrast, increased levels of BCAAs are positively associated with a marker of insulin resistance, HOMA-IR (Matthews *et al*., 1985) in children (McCormack *et al*., 2013), with the future development of insulin resistance in young adults (Wurtz *et al*., 2013) and with HOMA-IR in middle-aged adults (Newgard *et al*., 2009). Furthermore, the first step in the degradation of glutathione produces γ-glutamylamino acids (Meister, 1973), which have been suggested to reflect decreased glutathione (Kalhan *et al*., 2011). Glutathione is decreased in older adults and, restoring glutathione to levels found in young subjects reduced HOMA-IR (Nguyen *et al*., 2013), evidence that collectively suggests a link between γ-glutamylamino acids with insulin sensitivity. Interestingly, glutathione is a tripeptide comprised of glutamate, cysteine and glycine, and the 6-month change in AA Factor 2, containing the amino acid glycine was positively associated with the 6-month change in SPPB, evidence that may additionally support a role for mechanisms related to glutathione status on influencing physical function in older adults.

In summary, our findings suggest that mechanisms related to gut bacterial metabolism, PPAR-α activation, and insulin sensitivity may underlie baseline physical function and improvements in function as a result of a combined resistance exercise and nutritional supplementation intervention in functionally-limited older adults. One limitation of our findings is that with the exception of isobutyrylcarnitine and N_6_-acetyllysine, metabolites that were associated with function at baseline were not associated with the 6-month change in function. While this suggests that mechanisms underlying baseline and the 6-month change in function are different, it is important to note that associations between (albeit different) metabolites with gut bacterial metabolism, PPAR-α activation, and insulin sensitivity were associated with both baseline and the 6-month change in function. For example, whereas BCAAs were negatively associated with baseline 400-m but not with measures of function after 6 months of the combined resistance exercise and nutritional supplementation intervention, in contrast, γ-glutamylBCAAs were negatively associated with the 6-month change in LP/Lean, evidence that suggests a link between BCAA-related metabolism with both baseline and the 6-month change in function. Separately, although use of regression-based predictive modeling identified combinations of PCA factors that explained between 7% and 34% (avg., 18.5%) of the variance inherent in baseline and the 6-month change in function, we acknowledge that because of our relatively small sample size, validation of these results in a larger cohort are necessary. Collectively, results obtained in the current study suggest that future studies aimed at testing the causative role of gut bacteria-related metabolites, PPAR-α activation, and insulin sensitivity on influencing physical function in functionally-limited older adults are of interest.

## Experimental procedures

### Subjects

Inclusion and exclusion criteria and results for the LP one repetition maximum (measured with K400; Keiser Sports Health Equipment Inc., Fresno, CA, USA), SPPB, 400-m, total lean and fat mass (measured by dual-energy X-ray absorptiometry), BMI, dietary protein and fiber intake in older adults with a demonstrated reduction in mobility (SPPB≤10) from the randomized, double-blind, controlled study of (Chale *et al*., 2012) were used. Briefly, study participants were required to be sedentary at baseline, defined as the absence of structured exercise during the previous 6 months. All subjects then underwent a supervised program of resistance exercise training, three times per week for 6 months. With the goal of improving gains in muscle mass and physical function as a result of resistance exercise training, half of the study participants consumed 40 g day^−1^ of a whey protein-containing supplement; the remaining subjects consumed placebo. Serum was obtained at baseline (*N* = 73, 43 women, 30 men) and after 6 months of the combined resistance exercise and nutritional supplementation intervention (SPPB, 400-m, *N* = 67, 42 women, 25 men; LP/Lean, *N* = 64, 40 women, 24 men) for use in metabolomic analyses. The study was approved by the Tufts University Health Sciences Campus Institutional Review Board.

### Measurement of serum creatinine

Ten milliliters of blood was collected under standardized conditions, between 8 and 10 am, and following an overnight fast. After 6 months of the combined resistance exercise and nutritional supplementation intervention, blood was collected 48 h following the last exercise session. After collection, blood was allowed to clot for 1 h at room temperature and centrifuged at 2135 *g* for 10 min at 4 °C. Serum was derived by removing the supernatant following centrifugation and was stored in 1 mL aliquots at −80 °C, prior to analysis. Creatinine was measured using a clinical chemistry automated analyzer (Olympus AU400; Olympus America Inc., Melville, NY, USA) using reagents, calibrators, and standard operating procedures as specified by the manufacturer.

### Metabolomic analysis

Serum metabolomic data acquisition was performed by Metabolon Inc. (Research Triangle Park, NC, USA) as previously reported (Evans *et al*., 2009). Briefly, small molecule metabolites were extracted from serum, and the reconstituted extracts were resolved using MS platforms, including ultrahigh performance liquid chromatography/tandem mass spectrometry (UHPLC/MS/MS) and gas chromatography/mass spectrometry (GC/MS). Serum metabolomic data for amino acids, fatty acids, and acylcarnitines have previously been used to improve understanding about conditions related to alterations in oxidative capacity (Newgard *et al*., 2009). With use of MS-based metabolomics, relative quantitative data for 296 metabolites were identified. Of these, 177 metabolites, including 98 amino acids, 59 fatty acids, and 20 acylcarnitines, were then used in statistical analyses.

### Statistics

Given the large number of predictors (177 metabolites) and potential collinearity of metabolites, PCA was performed to decrease these multiple metabolites into a smaller set of constructed latent variables (principal components or ‘factors’) and, to control for type I error, as previously reported in studies of metabolic intermediates (Huffman *et al*., 2009). PCA-obtained factors account for common variance in the original measurements, with metabolites that correlate highly with one another forming common factors. Principal components analysis was performed separately for amino acids, fatty acids, and acylcarnitines using orthogonal varimax rotation (SAS Enterprise Guide 4.3; SAS Institute Inc., Cary, NC, USA). To account for the sample size at baseline (*N* = 73), PCA was performed on two separate amino acid-containing groups, one including 72 metabolites and the other containing primarily dipeptides (26 metabolites). Components with an eigenvalue > 1 were retained, resulting in 21, 13, 7, and 6 metabolite-containing, PCA-derived baseline factors for amino acids, fatty acids, dipeptides, and acylcarnitines, respectively. Individual metabolites with a component loading of 0.4 or greater were considered as being contained within each factor. Subtraction of baseline MS data from the 6-month MS data was used to determine 6-month change PCA factors. To account for the 6-month change in SPPB and 400-m sample size (*N* = 67), PCA was performed on two separate amino acid-containing groups, including 66 and 32 metabolites, respectively. Additionally, to account for the 6-month change in LP/Lean sample size (*N* = 64), PCA was performed on two separate amino acid-containing groups, including 63 and 35 metabolites, respectively. Use of PCA resulted in identification of 22, 13, 11, and 6 six-month change PCA factors for amino acids, fatty acids, dipeptides, and acylcarnitines that were used in 6-month change in SPPB and 400-m analyses and 21, 13, 12, and 5 six-month change PCA factors that were used for the 6-month change in LP/Lean analysis. Following PCA, factor scores for each subject were computed using SAS Enterprise Guide 4.3, as previously reported (Huffman *et al*., 2009).

Multivariable-adjusted linear regression (SAS Enterprise Guide 4.3) was used to examine the association between PCA factors with measures of physical function. At baseline, each model included sex, age, total fat (for the LP/Lean model) or BMI (SPPB and 400-m models), and each respective PCA factor. Linear regression models that investigated the association between the 6-month change in LP/Lean, SPPB, and 400-m with the 6-month change in PCA factors included sex, age, 6-month fat change (for the 6-month change in LP/Lean model) or 6-month BMI change (for the 6-month change in SPPB and 400-m models), whey/placebo group designation, and baseline function (LP/Lean, SPPB or 400-m, respectively). False discovery rates (Benjamini & Hochberg, 1995) were computed with use of the *q*-value method (Storey & Tibshirani, 2003) to account for multiple comparisons. Statistical significance for multivariable-adjusted associations between PCA factors with measures of physical function was set at *P* ≤ 0.05 and *q* ≤ 0.30, as reported by (Meyers *et al*., 2010). A *q*-value of 0.30 indicates that the result is likely to be valid seven of 10 times, which we suggest is reasonable in the setting of exploratory discovery.

Inclusion of covariates with *P*-values <0.05 in regression-based predictive modeling can fail in identifying variables known to be important (Mickey & Greenland, 1989). Therefore, PCA factors that were associated with measures of physical function at *P* ≤ 0.10 [as recommended by Bursac *et al*. (2008)] and *q* ≤ 0.30 in multivariable-adjusted linear regression analyses were considered as candidate variables for BE regression, with the goal of identifying a metabolite predictor set representative of baseline and the 6-month change in LP/Lean, SPPB, and 400-m. This cut point was chosen to permit the BE process enough flexibility so that PCA factors containing suggestive associations with measures of physical function could be retained in the final model (Lewis *et al*., 2013). At baseline, BE models included sex, age, total fat (for the LP/Lean model) or BMI (SPPB and 400-m models), and PCA factors that were associated (*P* ≤ 0.10 and *q* ≤ 0.30) with LP/Lean, SPPB, or 400-m as candidate variables. BE models that investigated the association between the 6-month change in PCA factors with the 6-month change in LP/Lean, SPPB, and 400-m included sex, age, 6-month fat change (for the 6-month change in LP/Lean model) or 6-month BMI change (for the 6-month change in SPPB and 400-m models), whey/placebo group designation, and baseline function (LP/Lean, SPPB, or 400-m, respectively) as candidate variables. Statistical significance to be retained in BE models was set at *P* < 0.10 (Bursac *et al*., 2008).

An increased dietary protein intake or reduced kidney function may result in an increased serum concentration of amino acid-related degradation products (Burke *et al*., 2012; Boelaert *et al*., 2013). Therefore, PCA factors that were significantly associated with measures of physical function in multivariable-adjusted or, in BE models, and that have been previously found in the serum or colonic lumen from conventional, but not germ-free mice (Wikoff *et al*., 2009; Matsumoto *et al*., 2012) including AA Factors 6, 7, 12, 13 and the 6-month change in FA Factor 5 and AA Factors 5, 8, 12, and 17 were further examined for their association with baseline and the 6-month change in average dietary protein intake (*N* = 67, baseline; *N* = 55 and *N* = 52 for the 6-month change in PCA factors associated with the 6-month change in SPPB and 400-m and LP/Lean, respectively) using the 3-day diet records of (Chale *et al*., 2012) and serum creatinine (*N* = 73, baseline; *N* = 63 and *N* = 61 for the 6-month change in PCA factors associated with the 6-month change in SPPB and 400-m and LP/Lean, respectively). Linear regression models that examined the association between dietary protein intake and serum creatinine with gut bacterial-related PCA factors at baseline were adjusted for sex, age, and total fat or BMI, depending on whether the PCA factor was associated with LP/Lean or SPPB and 400-m. The 6-month change in dietary protein intake or serum creatinine was examined for its association with significant 6-month change in gut bacterial-related PCA factors and included adjustment for sex, age, whey/placebo group designation, 6-month fat or BMI change (depending on whether the PCA factor was associated with LP/Lean or SPPB and 400-m) and baseline dietary protein intake or creatinine, respectively. Separately, because fermentation of dietary fiber by colonic bacteria results in the production of short chain fatty acids, including acetate (Cummings, 1983), the sex, age, and BMI-adjusted association between average dietary fiber intake [*N* = 66 at baseline, obtained from the 3-day diet records of (Chale *et al*., 2012)] with *N*-acetylglycine [glycine conjugation with acetyl-CoA yields *N*-acetylglycine (Schachter & Taggart, 1954)] was examined.

## Supporting Information

**Table S1.** Associations between PCA Factors with LP/Lean, SPPB and 400-m at baseline. **Table S2.** Associations between dietary protein intake or serum creatinine with gut-bacterial related PCA Factors that were significantly associated with physical function at baseline. **Table S3.** Associations between the 6-month change in PCA Factors with the 6-month change in LP/Lean. **Table S4.** Associations between the 6-month change in PCA Factors with the 6-month change in SPPB and 400-m. **Table S5.** Associations between the 6-month change in dietary protein intake or serum creatinine with the 6-month change in gut-bacterial related PCA Factors that were significantly associated with physical function after six-months of the combined resistance exercise and nutritional supplementation intervention.
