# Supplementary material for: Metabolites related to gut bacterial metabolism, peroxisome proliferator-activated receptor-alpha activation, and insulin sensitivity are associated with physical function in functionally-limited older adults
Source: Aging Cell. 2014 Jul 18;13(5):918–25. doi: 10.1111/acel.12251 (PMC4331755; doi:10.1111/acel.12251)
Supplement: Supplementary file 1 — Table S1. Associations between PCA Factors with LP/Lean, SPPB and 400-m at baseline. Table S2. Associations between dietary protein intake or serum creatinine with gut-bacterial related PCA Factors that were significantly associated with physical function at baseline. Table S3. Associations between the 6-month change in PCA Factors with the 6-month change in LP/Lean. Table S4. Associations between the 6-month change in PCA Factors with the 6-month change in SPPB and 400-m. Table S5. Associations between the 6-month change in dietary protein intake or serum creatinine with the 6-month change in gut-bacterial related PCA Factors that were significantly associated with physical function after six-months of the combined resistance exercise and nutritional supplementation intervention. [file acel0013-0918-sd1.docx]

**Supplementary Table S1**. Associations between PCA Factors with LP/Lean, SPPB and 400-m at baseline.

|  |  | **LP/Lean** | | | **SPPB** | | | **400-m** | | |
| --- | --- | --- | --- | --- | --- | --- | --- | --- | --- | --- |
|  | **Component Loadings** | **Β ± SE** | **p-value** | **q-value** | **Β ± SE** | **p-value** | **q-value** | **Β ± SE** | **p-value** | **q-value** |
| **AC F1:** octanoylcarnitine, decanoylcarnitine, cis-decenoylcarnitine, haxanoylcarnitine, lauroylcarnitine | 0.96, 0.92, 0.90, 0.86, 0.45 | 0.1 ± 0.7 | 0.91 | 0.32 | -0.0 ± 0.2 | 0.79 | 0.32 | 0.0 ± 0.0 | 0.29 | 0.32 |
| **AC F2:** palmitoylcarnitine, oleoylcarnitine, lauroylcarnitine | 0.96, 0.95, 0.51 | -0.5 ± 0.7 | 0.48 | 0.32 | -0.1 ± 0.2 | 0.68 | 0.32 | -0.0 ± 0.0 | 0.73 | 0.32 |
| **AC F3:** isovalerylcarnitine | 0.90 | 1.1 ± 0.7 | 0.14 | 0.32 | 0.2 ± 0.2 | 0.37 | 0.32 | -0.0 ± 0.0 | 0.52 | 0.32 |
| **AC F4:** isobutyrylcarnitine | 0.91 | -0.2 ± 0.7 | 0.74 | 0.32 | -0.2 ± 0.2 | 0.39 | 0.32 | -0.0 ± 0.0 | 0.02 | 0.25 |
| **AC F5:** tiglylcarnitine | 0.92 | 0.4 ± 0.7 | 0.57 | 0.32 | -0.0 ± 0.2 | 0.92 | 0.32 | 0.0 ± 0.0 | 0.09 | 0.29 |
| **AC F6:** carnitine | 0.96 | 0.5 ± 0.7 | 0.52 | 0.32 | 0.0 ± 0.2 | 0.88 | 0.32 | 0.0 ± 0.0 | 0.48 | 0.32 |
| **FA F1:** margarate, 10-nonadecenoate, dihomo-linoleate, stearate, linoleate, oleate, palmitate, eicosenoate, nonadecanoate, 10-heptadecenoate, docosadienoate, myristate, palmitoleate, 17-methylstearate, pentadecanoate, 15-methylpalmitate, linolenate, cis-vaccenate, adrenate, dihomo-linolenate, docosapentaenoate, myristoleate | 0.91, 0.89, 0.87, 0.86, 0.85, 0.85, 0.84, 0.84, 0.83, 0.81, 0.78, 0.73, 0.68, 0.65, 0.65, 0.60, 0.60, 0.55, 0.52, 0.50, 0.48, 0.44 | 0.9 ± 0.7 | 0.21 | 0.32 | 0.0 ± 0.2 | 0.83 | 0.32 | 0.0 ± 0.0 | 0.49 | 0.32 |
| **FA F2:** azelate, undecanedioate, sebacate, suberate, adipate, 8-hydroxyoctanoate, dodecanedioate | 0.97, 0.96, 0.94, 0.90, 0.76, 0.74, 0.44 | -0.2 ± 0.7 | 0.76 | 0.32 | -0.1 ± 0.2 | 0.54 | 0.32 | -0.0 ± 0.0 | 0.90 | 0.32 |
| **FA F3:** caprylate, heptanoate, pelargonate, caproate, caprate, undecanoate | 0.91, 0.87, 0.85, 0.79, 0.67, 0.42 | -0.3 ± 0.7 | 0.67 | 0.32 | -0.1 ± 0.2 | 0.51 | 0.32 | 0.0 ± 0.0 | 0.59 | 0.32 |
| **FA F4:** docosahexaenoate, eicosapentaenoate, docosapentaenoate, stearidonate, 3-carboxy-4-methyl-5-propyl-2-furanpropanoate | 0.93, 0.93, 0.72, 0.59, 0.52 | -0.2 ± 0.7 | 0.81 | 0.32 | 0.2 ± 0.2 | 0.20 | 0.32 | -0.0 ± 0.0 | 0.90 | 0.32 |
| **FA F5:** 3-hydroxydecanoate, 3-hydroxylaurate, 3-hydroxyoctanoate | 0.87, 0.78, 0.78 | -0.4 ± 0.7 | 0.57 | 0.32 | 0.0 ± 0.2 | 0.84 | 0.32 | 0.0 ± 0.0 | 0.30 | 0.32 |
| **FA F6:** tetradecanedioate, hexadecanedioate, dodecanedioate, octadecanedioate | 0.88, 0.85, 0.46, 0.44 | -1.0 ± 0.7 | 0.14 | 0.32 | 0.1 ± 0.2 | 0.58 | 0.32 | -0.0 ± 0.0 | 0.24 | 0.32 |
| **FA F7:** 5-dodecenoate, myristoleate, palmitoleate | 0.79, 0.78, 0.48 | 0.8 ± 0.8 | 0.28 | 0.32 | -0.2 ± 0.2 | 0.29 | 0.32 | 0.0 ± 0.0 | 0.03 | 0.25 |
| **FA F8:** valerate | 0.89 | -0.1 ± 0.7 | 0.87 | 0.32 | -0.0 ± 0.2 | 0.84 | 0.32 | -0.0 ± 0.0 | 0.39 | 0.32 |
| **FA F9:** 2-hydroxypalmitate, 2-hydroxystearate | 0.90, 0.42 | 1.5 ± 0.7 | 0.03 | 0.25 | 0.1 ± 0.2 | 0.70 | 0.32 | 0.0 ± 0.0 | 0.72 | 0.32 |
| **FA F10:** palmitate, methyl ester | 0.97 | 0.5 ± 0.7 | 0.53 | 0.32 | -0.1 ± 0.2 | 0.72 | 0.32 | 0.0 ± 0.0 | 0.47 | 0.32 |
| **FA F11:** erucamide | 0.96 | -0.0 ± 0.7 | 0.97 | 0.32 | 0.1 ± 0.2 | 0.56 | 0.32 | -0.0 ± 0.0 | 0.72 | 0.32 |
| **FA F12:** 13-HODE + 9-HODE | 0.82 | -0.3 ± 0.7 | 0.71 | 0.32 | 0.1 ± 0.2 | 0.50 | 0.32 | 0.0 ± 0.0 | 0.53 | 0.32 |
| **FA F13:** 10-undecenoate | 0.92 | 0.4 ± 0.7 | 0.55 | 0.32 | -0.1 ± 0.2 | 0.50 | 0.32 | 0.0 ± 0.0 | 0.09 | 0.29 |
| **AA F1:** 2-hydroxyisobutyrate, N-acetylthreonine, urea, C-glycosyltryptophan, N-acetylalanine, N-formylmethionine, serine (-), β-hydroxyisovalerate, N_6_-acetyllysine | 0.90, 0.79, 0.74, 0.70, 0.58, 0.48, -0.46, 0.44, 0.42 | 0.2 ± 0.7 | 0.79 | 0.32 | -0.1 ± 0.2 | 0.41 | 0.32 | -0.0 ± 0.0 | 0.06 | 0.29 |
| **AA F2:** 4-methyl-2-oxopentanoate, 3-methyl-2-oxobutyrate, 3-methyl-2-oxovalerate, 3-hydroxyisobutyrate | 0.87, 0.83, 0.83, 0.41 | 1.0 ± 0.7 | 0.16 | 0.32 | -0.0 ± 0.2 | 0.93 | 0.32 | 0.0 ± 0.0 | 0.21 | 0.32 |
| **AA F3:** aspartate, 5-oxoproline, glutamate, serine | 0.90, 0.83, 0.65, 0.57 | 0.3 ± 0.7 | 0.69 | 0.32 | -0.3 ± 0.2 | 0.13 | 0.32 | 0.0 ± 0.0 | 0.88 | 0.32 |
| **AA F4:** α-hydroxyisovalerate, 2-hydroxy-3-methylvalerate, α-hydroxyisocaproate, indolelactate | 0.87, 0.78, 0.67, 0.42 | 0.6 ± 0.7 | 0.40 | 0.32 | 0.1 ± 0.2 | 0.42 | 0.32 | 0.1 ± 0.0 | 0.02 | 0.25 |
| **AA F5:** valine, leucine, isoleucine | 0.80, 0.77, 0.76 | 0.1 ± 0.7 | 0.84 | 0.32 | 0.1 ± 0.2 | 0.67 | 0.32 | -0.0 ± 0.0 | 0.04 | 0.25 |
| **AA F6:** cinnamoylglycine, 3-phenylpropionate | 0.91, 0.89 | -1.5 ± 0.7 | 0.04 | 0.25 | -0.5 ± 0.2 | 0.009 | 0.25 | -0.0 ± 0.0 | 0.54 | 0.32 |
| **AA F7:** stachydrine, N-methyl proline | 0.92, 0.91 | -0.6 ± 0.7 | 0.36 | 0.32 | -0.4 ± 0.2 | 0.02 | 0.25 | 0.0 ± 0.0 | 0.59 | 0.32 |
| **AA F8:** S-methylcysteine, pipecolate | 0.88, 0.78 | -0.1 ± 0.7 | 0.93 | 0.32 | -0.2 ± 0.2 | 0.38 | 0.32 | 0.0 ± 0.0 | 0.96 | 0.32 |
| **AA F9:** methionine | 0.80 | 0.3 ± 0.7 | 0.69 | 0.32 | 0.1 ± 0.2 | 0.65 | 0.32 | 0.0 ± 0.0 | 0.86 | 0.32 |
| **AA F10:** homostachydrine | 0.94 | 1.1 ± 0.7 | 0.12 | 0.32 | -0.1 ± 0.2 | 0.45 | 0.32 | 0.0 ± 0.0 | 0.11 | 0.32 |
| **AA F11:** betaine | 0.89 | -0.1 ± 0.7 | 0.89 | 0.32 | -0.0 ± 0.2 | 0.86 | 0.32 | -0.0 ± 0.0 | 0.60 | 0.32 |
| **AA F12:** phenol sulfate | 0.91 | 0.2 ± 0.7 | 0.73 | 0.32 | -0.0 ± 0.2 | 0.91 | 0.32 | -0.0 ± 0.0 | 0.04 | 0.25 |
| **AA F13:** trans-urocanate | 0.92 | 0.1 ± 0.7 | 0.92 | 0.32 | 0.2 ± 0.2 | 0.36 | 0.32 | -0.0 ± 0.0 | 0.05 | 0.25 |
| **AA F14:** asparagine | 0.89 | 1.0 ± 0.7 | 0.20 | 0.32 | 0.1 ± 0.2 | 0.41 | 0.32 | 0.0 ± 0.0 | 0.60 | 0.32 |
| **AA F15:** beta-alanine | 0.86 | 0.2 ± 0.7 | 0.78 | 0.32 | -0.1 ± 0.2 | 0.77 | 0.32 | 0.0 ± 0.0 | 0.13 | 0.32 |
| **AA F16:** isovalerate | 0.85 | 0.8 ± 0.7 | 0.29 | 0.32 | 0.0 ± 0.2 | 0.83 | 0.32 | 0.0 ± 0.0 | 0.51 | 0.32 |
| **AA F17:** 3-methoxytyrosine | 0.90 | -0.1 ± 0.7 | 0.91 | 0.32 | -0.0 ± 0.2 | 0.92 | 0.32 | 0.0 ± 0.0 | 0.14 | 0.32 |
| **AA F18:** phenyllactate | 0.91 | 0.3 ± 0.7 | 0.65 | 0.32 | 0.1 ± 0.2 | 0.59 | 0.32 | 0.0 ± 0.0 | 0.88 | 0.32 |
| **AA F19:** N-acetylglycine | 0.92 | 1.2 ± 0.7 | 0.09 | 0.29 | 0.1 ± 0.2 | 0.77 | 0.32 | 0.1 ± 0.0 | 0.009 | 0.25 |
| **AA F20:** histidine | 0.87 | 0.6 ± 0.7 | 0.40 | 0.32 | 0.1 ± 0.2 | 0.76 | 0.32 | 0.0 ± 0.0 | 0.92 | 0.32 |
| **AA F21:** 4-oxovalerate | 0.92 | -0.6 ± 0.7 | 0.41 | 0.32 | 0.3 ± 0.2 | 0.13 | 0.32 | -0.0 ± 0.0 | 0.98 | 0.32 |
| **Dipept. F1:** γ**-**glutamylisoleucine, γ**-**glutamylvaline, γ-glutamylleucine, leucylleucine, γ-glutamyltyrosine, γ-glutamylthreonine, γ-glutamylphenylalanine | 0.92, 0.91, 0.90, 0.65, 0.540.46, 0.45 | 0.6 ± 0.7 | 0.40 | 0.32 | 0.1 ± 0.2 | 0.52 | 0.32 | 0.0 ± 0.0 | 0.54 | 0.32 |
| **Dipept. F2:** aspartylphenylalanine, phenylalanylphenylalanine, leucylleucine | 0.88, 0.72, 0.42 | 0.2 ± 0.7 | 0.75 | 0.32 | -0.3 ± 0.2 | 0.16 | 0.32 | -0.0 ± 0.0 | 0.93 | 0.32 |
| **Dipept. F3:** γ**-**glutamylglutamine, γ**-**glutamylalanine, γ**-**glutamylmethionine | 0.91, 0.81, 0.74 | -1.2 ± 0.7 | 0.09 | 0.29 | -0.1 ± 0.2 | 0.53 | 0.32 | 0.0 ± 0.0 | 0.78 | 0.32 |
| **Dipept. F4:** leucylphenylalanine, isoleucylvaline | 0.90, 0.82 | -0.4 ± 0.7 | 0.59 | 0.32 | -0.1 ± 0.2 | 0.63 | 0.32 | -0.0 ± 0.0 | 0.22 | 0.32 |
| **Dipept. F5:** phenylalanylserine | 0.91 | 0.9 ± 0.7 | 0.20 | 0.32 | 0.3 ± 0.2 | 0.11 | 0.32 | 0.0 ± 0.0 | 0.75 | 0.32 |
| **Dipept. F6:** citrulline | 0.95 | 0.8 ± 0.7 | 0.24 | 0.32 | -0.0 ± 0.2 | 0.85 | 0.32 | 0.0 ± 0.0 | 0.59 | 0.32 |
| **Dipept. F7:** serylleucine | 0.97 | 0.1 ± 0.7 | 0.86 | 0.32 | -0.1 ± 0.2 | 0.49 | 0.32 | -0.0 ± 0.0 | 0.29 | 0.32 |

Sex, age, total fat (LP/Lean model) or BMI (SPPB, 400-m models)-adjusted associations between PCA factors with LP/Lean, SPPB and 400-m at baseline are shown with component loadings for metabolites≥0.4, parameter estimates and standard errors (β ± SE), in order of significance (p-value) and, with q-values.

**Supplementary Table S2.** Associations between dietary protein intake or serum creatinine with gut-bacterial related PCA Factors that were significantly associated with physical function at baseline.

|  | **Dietary Protein** | **p-value** | **Serum Creatinine** | **p-value** |
| --- | --- | --- | --- | --- |
| AA Factor 6 (LP/Lean model) | 2.7 ± 1.8 | 0.14 | 0.0 ± 0.0 | 0.77 |
| AA Factor 6 (SPPB model) | 2.6 ± 1.8 | 0.16 | 0.0 ± 0.0 | 0.67 |
| AA Factor 7 | -2.7 ± 1.8 | 0.15 | 0.0 ± 0.0 | 0.33 |
| AA Factor 12 | -1.6 ± 1.9 | 0.40 | 0.0 ± 0.0 | 0.24 |
| AA Factor 13 | -2.6 ± 1.9 | 0.18 | 0.0 ± 0.0 | 0.92 |

Associations between PCA Factors with dietary protein intake and serum creatinine are shown with parameter estimates and standard errors (β ± SE), and, p-values.

**Supplementary Table S3.** Associations between the six-month change in PCA Factors with the six-month change in LP/Lean.

|  |  | **Six Month Change, LP/Lean** | | |
| --- | --- | --- | --- | --- |
|  | **Component Loadings** | **Β ± SE** | **p-value** | **q-value** |
| **AC F1:** octanoylcarnitine, decanoylcarnitine, cis-decenoylcarnitine, hexanoylcarnitine, acetylcarnitine | 0.95, 0.89, 0.87, 0.82, 0.55 | 0.2 **±** 0.7 | 0.84 | 0.32 |
| **AC F2:** palmitoylcarnitine, oleoylcarnitine, lauroylcarnitine | 0.95, 0.90, 0.52 | -0.4 ± 0.7 | 0.57 | 0.32 |
| **AC F3:** isobutyrylcarnitine | 0.94 | 0.8 ± 0.7 | 0.27 | 0.32 |
| **AC F4:** glutarylcarnitine | 0.94 | 1.5 ± 0.7 | 0.05 |  |
| **AC F5:** carnitine | 0.93 | -0.4 ± 0.7 | 0.54 | 0.25 |
| **FA F1:** 10-heptadecenoate**,** linoleate, palmitoleate, oleate, margarate, 10-nonadecenoate, palmitate, linolenate, dihomo-linoleate, eicosenoate, myristate, nonadecanoate, stearate, docosapentaenoate, docosadienoate, myristoleate, cis-vaccenate, dihomo-linolenate, hexadecanedioate, 17-methylstearate, 5-dodecenoate, adrenate, 3-hydroxydecanoate, tetradecanedioate, 15-methylpalmitate, laurate, pentadecanoate, stearidonate, octadecanedioate, eicosapentaenoate, arachidonate, 3-hydroxylaurate, docosahexanoate | 0.95, 0.94, 0.94, 0.93, 0.92, 0.90, 0.90, 0.90, 0.89, 0.89, 0.86, 0.81, 0.81, 0.79, 0.79, 0.78, 0.78, 0.70, 0.66, 0.65, 0.65, 0.64, 0.61, 0.60, 0.57, 0.53, 0.53, 0.53, 0.51, 0.51, 0.51, 0.45, 0.43 | 1.1 ± 0.7 | 0.14 | 0.32 |
| **FA F2:** azelate, undecanedioate, sebacate, 8-hydroxyoctanoate, suberate, adipate, dodecanedioate, 2-hydroxyglutarate | 0.98, 0.95, 0.95, 0.76, 0.73, 0.55, 0.46, 0.44 | 0.1 ± 0.7 | 0.91 | 0.32 |
| **FA F3:** caprylate, pelargonate, heptanoate, caprate, caproate | 0.93, 0.93, 0.84, 0.80, 0.78 | 0.1 ± 0.7 | 0.92 | 0.32 |
| **FA F4:** docosahexaenoate, eicosapentaenoate, docosapentaenoate | 0.80, 0.53, 0.49 | 0.6 ± 0.7 | 0.44 | 0.32 |
| **FA F5:** valerate | 0.94 | 1.3 ± 0.7 | 0.07 | 0.29 |
| **FA F6:** 8-hydroxyoctanoate | 0.87 | 0.1 ± 0.7 | 0.94 | 0.32 |
| **FA F7:** undecanoate | 0.90 | -0.3 ± 0.7 | 0.67 | 0.32 |
| **FA F8:** 3-carboxy-4-methyl-5-propyl-2-furanpropanoate | 0.94 | -0.6 ± 0.7 | 0.39 | 0.32 |
| **FA F9:** n-butyl oleate | 0.95 | -0.1 ± 0.7 | 0.95 | 0.32 |
| **FA F10:** 7-hydroxyoctanoate | 0.96 | -0.2 ± 0.7 | 0.79 | 0.32 |
| **FA F11:** docosapentaenoate | 0.86 | -0.2 ± 0.7 | 0.79 | 0.32 |
| **FA F12:** erucamide | 0.94 | 0.5 ± 0.7 | 0.53 | 0.32 |
| **FA F13:** palmitate, methyl ester | 0.98 | 0.4 ± 0.7 | 0.61 | 0.32 |
| **AA F1:** leucine, tyrosine, phenylalanine, valine, isoleucine, tryptophan | 0.86, 0.82, 0.75, 0.69, 0.65, 0.57 | 0.4 ± 0.7 | 0.57 | 0.32 |
| **AA F2:** glycine, serine, asparagine, alanine, glutamine | 0.90, 0.79, 0.72, 0.56, 0.53 | 0.2 ± 0.8 | 0.75 | 0.32 |
| **AA F3:** 4-methyl-2-oxopentanoate, 3-methyl-2-oxovalerate, 3-methyl-2-oxobutyrate | 0.86, 0.80, 0.80 | -0.1 ± 0.8 | 0.92 | 0.32 |
| **AA F4:** α**-**hydroxyisovalerate, indolelactate | 0.81, 0.76 | -0.1 ± 0.8 | 0.85 | 0.32 |
| **AA F5:** p-cresol sulfate, 3-indoxyl sulfate | 0.87, 0.84 | -0.1 ± 0.7 | 0.91 | 0.32 |
| **AA F6:** 3-phenylpropionate, cinnamoylglycine | 0.91, 0.79 | -0.3 ± 0.7 | 0.72 | 0.32 |
| **AA F7:** C-glycosyltryptophan | 0.89 | -0.1 ± 0.8 | 0.90 | 0.32 |
| **AA F8:** 3-(4-hydroxyphenyl)lactate | 0.88 | 0.1 ± 0.7 | 0.86 | 0.32 |
| **AA F9:** indoleacetate | 0.93 | 0.4 ± 0.7 | 0.58 | 0.32 |
| **AA F10:** imidazole propionate | 0.91 | 0.8 ± 0.7 | 0.31 | 0.32 |
| **AA F11:** aspartate | 0.84 | 0.1 ± 0.8 | 0.8 | 0.32 |
| **AA F12:** N-acetylthreonine | 0.90 | 0.4 ± 0.8 | 0.57 | 0.32 |
| **AA F13:** N6-acetyllysine | 0.93 | -1.4 ± 0.7 | 0.05 | 0.25 |
| **AA F14:** N-acetyl-beta-alanine | 0.93 | 0.5 ± 0.7 | 0.50 | 0.32 |
| **AA F15:** isovalerate | 0.87 | -0.7 ± 0.7 | 0.33 | 0.32 |
| **AA F16:** 3-hydroxy-2-ethylpropionate | 0.92 | -0.6 ± 0.7 | 0.44 | 0.32 |
| **AA F17:** 4-oxovalerate | 0.91 | -0.7 ± 0.7 | 0.34 | 0.32 |
| **AA F18:** homostachydrine | 0.92 | -0.2 ± 0.7 | 0.84 | 0.32 |
| **AA F19:** serotonin | 0.95 | -1.0 ± 0.7 | 0.20 | 0.32 |
| **AA F20:** glutamate | 0.89 | -0.7 ± 0.7 | 0.37 | 0.32 |
| **AA F21:** tryptophan betaine | 0.89 | -0.5 ± 0.7 | 0.48 | 0.32 |
| **Dipept. F1:** γ-glutamylleucine, γ**-**glutamylisoleucine, γ**-**glutamylvaline, γleucylleucine | 0.90, 0.88, 0.86, 0.47 | -1.4 ± 0.7 | 0.07 | 0.29 |
| **Dipept. F2:** γ**-**glutamylalanine, γ**-**glutamylglutamine, γ**-**glutamylmethionine | 0.93, 0.92, 0.60 | 0.3 ± 0.7 | 0.69 | 0.32 |
| **Dipept. F3:** phenylalanylleucine, aspartylphenylalanine, leucylalanine | 0.91, 0.87, 0.59 | -0.1 ± 0.7 | 0.94 | 0.32 |
| **Dipept. F4:** N-methyl proline, stachydrine | 0.94, 0.83 | 0.5 ± 0.7 | 0.54 | 0.32 |
| **Dipept. F5:** serylleucine | 0.92 | 0.6 ± 0.8 | 0.45 | 0.32 |
| **Dipept. F6:** leucylphenylalanine | 0.90 | 0.3 ± 0.8 | 0.7 | 0.32 |
| **Dipept. F7:** γ**-**glutamylphenylalanine | 0.93 | 0.2 ± 0.7 | 0.83 | 0.32 |
| **Dipept. F8:** N-acetylmethionine | 0.96 | -1.3 ± 0.7 | 0.08 | 0.29 |
| **Dipept. F9:** phenylalanylserine | 0.92 | -0.2 ± 0.8 | 0.82 | 0.32 |
| **Dipept. F10:** γ**-**glutamyltyrosine | 0.94 | -0.4 ± 0.8 | 0.60 | 0.32 |
| **Dipept. F11:** γ**-**glutamylthreonine | 0.94 | -0.3 ± 0.8 | 0.69 | 0.32 |
| **Dipept. F12:** trans-4-hydroxyproline | 0.94 | -0.4 ± 0.8 | 0.57 | 0.32 |

The six-month change in PCA factors that are associated with the six-month change in LP/Lean after adjusting for sex, age, six-month fat change, whey/placebo group designation, and baseline LP/Lean are shown with component loadings for metabolites≥0.4, parameter estimates and standard errors (β ± SE), in order of significance (p-value) and, with q-values.

**Supplementary Table S4.** Associations between the six-month change in PCA Factors with the six-month change in SPPB and 400-m.

|  |  | **Six-Month Change, SPPB** | | | **Six-Month Change, 400-m** | | |
| --- | --- | --- | --- | --- | --- | --- | --- |
|  | **Component Loadings** | **Β ± SE** | **p-value** | **q-value** | **Β ± SE** | **p-value** | **q-value** |
| **AC F1:** octanoylcarnitine, decanoylcarnitine, cis-decenoylcarnitine, hexanoylcarnitine, acetylcarnitine | 0.95, 0.89, 0.87, 0.80, 0.49 | 0.0 ± 0.2 | 0.97 | 0.32 | -0.0 ± 0.0 | 0.24 | 0.32 |
| **AC F2:** palmitoylcarnitine, oleoylcarnitine, lauroylcarnitine | 0.94, 0.90, 0.53 | -0.2 ± 0.2 | 0.36 | 0.32 | 0.0 ± 0.0 | 0.78 | 0.32 |
| **AC F3:** isovalerylcarnitine | 0.93 | 0.0 ± 0.2 | 0.82 | 0.32 | -0.0 ± 0.0 | 0.36 | 0.32 |
| **AC F4:** glutarylcarnitine | 0.94 | 0.1 ± 0.2 | 0.45 | 0.32 | -0.0 ± 0.0 | 0.93 | 0.32 |
| **AC F5:** isobutyrylcarnitine | 0.93 | 0.1 ± 0.2 | 0.55 | 0.32 | 0.1 ± 0.0 | 0.03 | 0.25 |
| **AC F6:** carnitine | 0.93 | -0.0 ± 0.2 | 0.84 | 0.32 | 0.0 ± 0.0 | 0.49 | 0.32 |
| **FA F1:** 10-heptadecenoate**,** linoleate, palmitoleate, oleate, margarate, 10-nonadecenoate, palmitate, linolenate, dihomo-linoleate, 10-nonadecanoate, eicosenoate, myristate, docosapentaenoate, stearate, nonadecanoate, myristoleate, cis-vaccenate, docosadienoate, dihomo-linolenate, adrenate, 5-dodecenoate, hexadecanedioate, 17-methylstearate, 3-hydroxydecanoate, tetradecanedioate, 15-methylpalmitate, stearidonate, pentadecanoate, arachidonate, eicosapentaenoate, laurate, octadecanedioate, 3-hydroxylaurate, docosahexanoate | 0.95, 0.94, 0.94, 0.93, 0.92, 0.90, 0.90, 0.90, 0.90, 0.90, 0.88, 0.86, 0.80, 0.80, 0.79, 0.79, 0.78, 0.76, 0.71, 0.66, 0.65, 0.63, 0.63, 0.60, 0.60, 0.57, 0.55, 0.54, 0.53, 0.52, 0.51, 0.48, 0.45, 0.45 | -0.3 ± 0.2 | 0.12 | 0.32 | 0.0 ± 0.0 | 0.48 | 0.32 |
| **FA F2:** azelate, sebacate, undecanedioate, 8-hydroxyoctanoate, suberate, adipate, dodecanedioate, 2-hydroxyglutarate | 0.97, 0.95, 0.94, 0.76, 0.72, 0.53, 0.43, 0.42 | -0.2 ± 0.2 | 0.26 | 0.32 | -0.0 ± 0.0 | 0.60 | 0.32 |
| **FA F3:** caprylate, pelargonate, heptanoate, caprate, caproate | 0.92, 0.92, 0.84, 0.80, 0.78 | -0.2 ± 0.2 | 0.24 | 0.32 | 0.0 ± 0.0 | 0.70 | 0.32 |
| **FA F4:** docosahexaenoate, eicosapentaenoate, docosapentaenoate | 0.79, 0.53, 0.48 | 0.3 ± 0.2 | 0.13 | 0.32 | 0.0 ± 0.0 | 0.57 | 0.32 |
| **FA F5:** valerate | 0.93 | -0.0 ± 0.2 | 0.95 | 0.32 | 0.0 ± 0.0 | 0.89 | 0.32 |
| **FA F6:** 8-hydroxyoctanoate | 0.89 | -0.1 ± 0.2 | 0.66 | 0.32 | -0.0 ± 0.0 | 0.29 | 0.32 |
| **FA F7:** undecanoate | 0.90 | -0.1 ± 0.2 | 0.76 | 0.32 | 0.0 ± 0.0 | 0.43 | 0.32 |
| **FA F8:** 3-carboxy-4-methyl-5-propyl-2-furanpropanoate | 0.94 | 0.4 ± 0.2 | 0.01 | 0.25 | 0.0 ± 0.0 | 0.38 | 0.32 |
| **FA F9:** n-butyl oleate | 0.95 | 0.1 ± 0.2 | 0.37 | 0.32 | 0.0 ± 0.0 | 0.73 | 0.32 |
| **FA F10:** 7-hydroxyoctanoate | 0.96 | 0.1 ± 0.2 | 0.69 | 0.32 | 0.0 ± 0.0 | 0.46 | 0.32 |
| **FA F11:** erucamide | 0.94 | -0.1 ± 0.2 | 0.52 | 0.32 | -0.0 ± 0.0 | 0.55 | 0.32 |
| **FA F12:** 10-undecanoate | 0.90 | 0.1 ± 0.2 | 0.53 | 0.32 | 0.0 ± 0.0 | 0.64 | 0.32 |
| **FA F13:** docosapentaenoate | 0.85 | -0.1 ± 0.2 | 0.47 | 0.32 | 0.0 ± 0.0 | 0.48 | 0.32 |
| **AA F1:** leucine, tyrosine, phenylalanine, methionine, valine, isoleucine, tryptophan | 0.87, 0.78, 0.74, 0.71, 0.70, 0.69, 0.65 | -0.1 ± 0.2 | 0.53 | 0.32 | 0.0 ± 0.0 | 0.67 | 0.32 |
| **AA F2:** glycine, serine, asparagine, alanine, glutamine | 0.90, 0.79, 0.70, 0.53, 0.47 | 0.3 ± 0.2 | 0.08 | 0.29 | -0.0 ± 0.0 | 0.47 | 0.32 |
| **AA F3:** 4-methyl-2-oxopentanoate, 3-methyl-2-oxobutyrate, 3-methyl-2-oxovalerate | 0.83, 0.83, 0.79 | -0.1 ± 0.2 | 0.67 | 0.32 | 0.0 ± 0.0 | 0.66 | 0.32 |
| **AA F4:** α**-**hydroxyisovalerate, indolelactate | 0.84, 0.67 | -0.1 ± 0.2 | 0.57 | 0.32 | 0.0 ± 0.0 | 0.62 | 0.32 |
| **AA F5:** p-cresol sulfate, 3-indoxyl sulfate | 0.88, 0.81 | -0.0 ± 0.2 | 0.97 | 0.32 | 0.1 ± 0.0 | 0.05 | 0.25 |
| **AA F6:** 3-phenylpropionate, cinnamoylglycine | 0.90, 0.81 | -0.0 ± 0.2 | 0.99 | 0.32 | -0.0 ± 0.0 | 0.19 | 0.32 |
| **AA F7:** C-glycosyltryptophan | 0.90 | -0.1 ± 0.2 | 0.44 | 0.32 | 0.0 ± 0.0 | 0.53 | 0.32 |
| **AA F8:** indoleacetate | 0.93 | 0.1 ± 0.2 | 0.56 | 0.32 | -0.1 ± 0.0 | 0.03 | 0.25 |
| **AA F9:** 4-oxovalerate | 0.90 | 0.1 ± 0.1 | 0.42 | 0.32 | 0.0 ± 0.0 | 0.85 | 0.32 |
| **AA F10:** aspartate | 0.83 | -0.2 ± 0.2 | 0.28 | 0.32 | -0.0 ± 0.0 | 0.20 | 0.32 |
| **AA F11:** N-acetylthreonine | 0.90 | -0.1 ± 0.2 | 0.78 | 0.32 | 0.0 ± 0.0 | 0.93 | 0.32 |
| **AA F12:** serotoin | 0.95 | 0.4 ± 0.2 | 0.03 | 0.25 | 0.0 ± 0.0 | 0.32 | 0.32 |
| **AA F13:** tryptophan betaine | 0.90 | 0.2 ± 0.2 | 0.19 | 0.32 | 0.0 ± 0.0 | 0.37 | 0.32 |
| **AA F14:** N-acetyl-beta-alanine | 0.93 | -0.3 ± 0.2 | 0.09 | 0.29 | -0.0 ± 0.0 | 0.34 | 0.32 |
| **AA F15:** N-acetylglycine | 0.91 | -0.2 ± 0.2 | 0.13 | 0.32 | -0.0 ± 0.0 | 0.48 | 0.32 |
| **AA F16:** N-formylmethionine | 0.93 | 0.2 ± 0.2 | 0.32 | 0.32 | 0.0 ± 0.0 | 0.24 | 0.32 |
| **AA F17:** dimethylglycine | 0.90 | -0.3 ± 0.2 | 0.05 | 0.25 | -0.0 ± 0.0 | 0.32 | 0.32 |
| **AA F18:** β-hydroxyisovalerate | 0.88 | 0.1 ± 0.2 | 0.43 | 0.32 | -0.0 ± 0.0 | 0.91 | 0.32 |
| **AA F19:** glutamate | 0.90 | 0.1 ± 0.2 | 0.55 | 0.32 | -0.0 ± 0.0 | 0.94 | 0.32 |
| **AA F20:** N_6_-acetyllysine | 0.91 | -0.2 ± 0.2 | 0.24 | 0.32 | -0.0 ± 0.0 | 0.18 | 0.32 |
| **AA F21:** homostachydrine | 0.92 | -0.0 ± 0.2 | 0.78 | 0.32 | 0.0 ± 0.0 | 0.42 | 0.32 |
| **AA F22:** isovalerate | 0.88 | -0.0 ± 0.2 | 0.87 | 0.32 | -0.0 ± 0.0 | 0.86 | 0.32 |
| **Dipept. F1:** γ-glutamylleucine, γ**-**glutamylisoleucine, γ**-**glutamylvaline, γleucylleucine | 0.90, 0.89, 0.84, 0.41 | -0.1 ± 0.2 | 0.45 | 0.32 | -0.0 ± 0.0 | 0.73 | 0.32 |
| **Dipept. F2:** γ**-**glutamylalanine, γ**-**glutamylglutamine, γ**-**glutamylmethionine | 0.93, 0.92, 0.62 | -0.1 ± 0.2 | 0.46 | 0.32 | -0.0 ± 0.0 | 0.85 | 0.32 |
| **Dipept. F3:** aspartylphenylalanine, phenylalanylleucine, leucylalanine | 0.90, 0.89, 0.60 | -0.1 ± 0.2 | 0.63 | 0.32 | -0.0 ± 0.0 | 0.26 | 0.32 |
| **Dipept. F4:** N-methyl proline, stachydrine | 0.95, 0.80 | 0.1 ± 0.2 | 0.50 | 0.32 | 0.0 ± 0.0 | 0.56 | 0.32 |
| **Dipept. F5:** serylleucine | 0.92 | 0.2 ± 0.2 | 0.29 | 0.32 | 0.0 ± 0.0 | 0.57 | 0.32 |
| **Dipept. F6:** phenylalanylserine | 0.92 | -0.1 ± 0.2 | 0.47 | 0.32 | -0.0 ± 0.0 | 0.21 | 0.32 |
| **Dipept. F7:** bradykinin | 0.96 | 0.1 ± 0.2 | 0.51 | 0.32 | 0.0 ± 0.0 | 0.87 | 0.32 |
| **Dipept. F8:** γ**-**glutamylphenylalanine | 0.94 | -0.0 ± 0.2 | 0.92 | 0.32 | 0.0 ± 0.0 | 0.96 | 0.32 |
| **Dipept. F9:** γ**-**glutamyltyrosine | 0.92 | -0.3 ± 0.2 | 0.04 | 0.32 | -0.0 ± 0.0 | 0.53 | 0.32 |
| **Dipept. F10:** leucylphenylalanine | 0.90 | -0.3 ± 0.2 | 0.09 | 0.29 | 0.0 ± 0.0 | 0.58 | 0.32 |
| **Dipept. F11:** homocitrulline | 0.95 | -0.1 ± 0.2 | 0.44 | 0.32 | 0.0 ± 0.0 | 0.20 | 0.32 |

The six-month change in PCA factors that are associated with the six-month change in SPPB and 400-m after adjusting for sex, age, six-month BMI change, whey/placebo group designation, and baseline SPPB or 400-m are shown with component loadings for metabolites≥0.4, parameter estimates and standard errors (β ± SE), in order of significance (p-value) and, with q-values.

**Supplementary Table S5.** Associations between the six-month change in dietary protein intake or serum creatinine with the six-month change in gut-bacterial related PCA. Factors that were significantly associated with physical function after six-months of the combined resistance exercise and nutritional supplementation intervention.

|  | **6-month Change in Dietary Protein** | **p-value** | **6-month Change in Serum Creatinine** | **p-value** |
| --- | --- | --- | --- | --- |
| FA Factor 5 | -0.5 ± 3.0 | 0.87 | 0.0 ± 0.0 | 0.38 |
| AA Factor 12 | -5.9 ± 3.0 | 0.06 | 0.0 ± 0.0 | 0.58 |
| AA Factor 17 | -1.2 ± 3.0 | 0.69 | 0.0 ± 0.0 | 0.56 |
| AA Factor 5 | 1.3 ± 2.9 | 0.65 | -0.0 ± 0.0 | 0.54 |
| AA Factor 8 | -2.2 ± 2.8 | 0.45 | -0.0 ± 0.0 | 0.51 |

Associations between the six-month change in PCA Factors with the six-month change in dietary protein intake and serum creatinine are shown with parameter estimates and standard errors (β ± SE), and, p-values.
